# ST8SIA6-AS1, a novel lncRNA star in liver cancer

**DOI:** 10.3389/fcell.2024.1435664

**Published:** 2024-08-15

**Authors:** Cheng Qiu, Haoran Fan, Siyu Tao, Ziqing Deng, Hongliang Luo, Fangteng Liu

**Affiliations:** ^1^ Department of General Surgery, Pingxiang People’s Hospital, Pingxiang, Jiangxi, China; ^2^ Department of Gastrointestinal Surgery, The Second Affiliated Hospital, Jiangxi Medical College, Nanchang University, Nanchang, Jiangxi, China; ^3^ Second School of Clinical Medicine, Jiangxi Medical College, Nanchang University, Nanchang, Jiangxi, China; ^4^ Department of General Surgery, Nanchang Third Hospital, Nanchang, Jiangxi, China

**Keywords:** liver cancer, ST8SIA6-AS1, tumorigenesis, tumor biomarker, therapeutic target

## Abstract

Liver cancer is one of the most lethal gastrointestinal malignancies. Emerging evidence has underscored the pivotal role of long non-coding RNAs (lncRNAs) in tumorigenesis, with ST8SIA6-AS1 identified as a novel oncogenic lncRNA contributing to liver cancer progression. ST8SIA6-AS1 is consistently upregulated in hepatic cancer tissues and is strongly associated with unfavorable prognosis. Moreover, it demonstrates high diagnostic efficacy in detecting HCC. ST8SIA6-AS1 is involved in various cellular processes including proliferation, migration, and invasion, primarily through its function as a competing endogenous RNA (ceRNA), thereby facilitating hepatocarcinogenesis and disease advancement. This review provides a detailed examination of the molecular functions and regulatory mechanisms of ST8SIA6-AS1 in hepatocellular carcinoma (HCC) and highlights its potential as a promising biomarker for liver cancer, aiming to propel the development of innovative therapeutic strategies for HCC management.

## 1 Introduction

Liver cancer, predominantly hepatocellular carcinoma (HCC), ranks among the most common and lethal cancers worldwide (Dhanasekaran et al., 2019; Llovet et al., 2021; Singal et al., 2023), particularly in regions plagued by endemic viral hepatitis (Wait et al., 2016; Ishizaki et al., 2017; Le et al., 2022; Shen et al., 2023). Despite advances in diagnostics and treatment, the prognosis for HCC patients remains dire (Villanueva et al., 2010; Ding and Wen, 2021), highlighting the critical need for more effective therapeutic strategies and biomarkers for early detection. Recent studies have increasingly highlighted the role of long non-coding RNAs (lncRNAs) in oncogenesis (Nandwani et al., 2021; Zhao et al., 2021; Liu et al., 2022; Chao et al., 2023; Silva et al., 2023; Coan et al., 2024). These lncRNAs, which do not encode proteins (Perkel, 2013; Mattick et al., 2023), are pivotal in regulating gene expression at various levels (Kung et al., 2013; Statello et al., 2021; Mangiavacchi et al., 2023; Ferrer and Dimitrova, 2024), including chromatin modification, transcription, and post-transcriptional processing. Among them, ST8 α-N-acetyl-neuraminide α-2,8-sialyltransferase six antisense RNA 1 (ST8SIA6-AS1), also known as APAL (Aurora A/Polo-like-kinase 1-associated lncRNA), has emerged as a significant contributor to the pathology of human cancers (Jeong et al., 2018; Huang CM. et al., 2020; Cao et al., 2020; Fang et al., 2020; Luo et al., 2020; Chen et al., 2021; He et al., 2021; Qiao et al., 2022; Wang et al., 2023), including liver cancer (Fei et al., 2020; Li and Jiang, 2020; Qin et al., 2020; Zhang et al., 2020; Zhang B. et al., 2021; Kuai et al., 2021; Mou and Ding, 2022; Feng et al., 2023).

*Homo sapiens* ST8SIA6-AS1 is a lncRNA gene situated on chromosome 10p12.33, comprising three exons and spanning 7,658 nucleotides (https://www.ncbi.nlm.nih.gov/gene/100506392). ST8SIA6-AS1 lncRNA is transcribed from the antisense strand of the ST8SIA6 gene and plays a pivotal role in various biological processes. Recently, ST8SIA6-AS1 has garnered attention due to its abnormal expression and oncogenic role in multiple types of tumor progression, including pituitary adenoma (Yin et al., 2021; Li et al., 2022), breast cancer (Jeong et al., 2018; Fang et al., 2020; Luo et al., 2020; Chen et al., 2021; Qiao et al., 2022), cholangiocarcinoma (He et al., 2021), lung cancer (Cao et al., 2020; Wang et al., 2023), and colorectal cancer (Huang CM. et al., 2020). Extensive research has highlighted the significant impact of ST8SIA6-AS1 in the development and progression of liver cancer (Fei et al., 2020; Li and Jiang, 2020; Qin et al., 2020; Zhang et al., 2020; Zhang B. et al., 2021; Kuai et al., 2021; Mou and Ding, 2022; Feng et al., 2023). It has been identified as a prognostic indicator for HCC patients, predictive of clinical features and outcomes (Luo et al., 2020; Zhang et al., 2020; Zhang B. et al., 2021; Zhang Y. et al., 2021; Xue et al., 2023). Moreover, serum ST8SIA6-AS1 is identified as a promising diagnostic biomarker for HCC (Qin et al., 2020). Recent studies also unravel the complex interactions of ST8SIA6-AS1 with other cellular machinery, implicating it in a broader spectrum of biological processes (Fei et al., 2020; Li and Jiang, 2020; Zhang et al., 2020; Zhang B. et al., 2021; Zhang Y. et al., 2021; Kuai et al., 2021; Mou and Ding, 2022; Feng et al., 2023; Xue et al., 2023), including proliferation, apoptosis and metastasis. These findings provide a foundation for hypothesizing that ST8SIA6-AS1 could serve as both a biomarker and a potential target for therapeutic intervention in liver cancer.

We conducted a comprehensive systematic search across multiple databases including Web of Science, PubMed, Embase, ScienceDirect, SpringerLink, and Google Scholar to compile relevant literature on the involvement of ST8SIA6-AS1 in liver cancer. Our search focused on studies published in peer-reviewed journals in English up to 1 June 2024, using keywords such as ‘ST8SIA6-AS1′, ‘APAL’, and ‘Aurora A/PLK1 Associated LncRNA’. The inclusion criteria were predefined to include original studies that investigated the expression, clinicopathological associations, and biological functions of ST8SIA6-AS1 in liver cancer.

In this review, we provide a comprehensive overview of the current knowledge on ST8SIA6-AS1 in liver cancer. We highlight its mechanistic roles in the progression of liver cancer, its potential as both a diagnostic and prognostic tool, and the opportunities it presents for targeted therapy in liver cancer. This review aims to examine the multifaceted roles of ST8SIA6-AS1 in cellular tumor processes and emphasize its significance as a promising lncRNA related to liver cancer. Additionally, we delineate both the current research gaps and future directions in this field.

## 2 ST8SIA6-AS1 expression in HCC and its involvement in tumorigenesis

ST8SIA6-AS1 is found to be significantly upregulated in various HCC cell lines when compared to normal human hepatocellular epithelial cells (Table 1). Notably, ST8SIA6-AS1 is predominantly localized in the cytoplasm of liver cancer cells, suggesting its involvement in post-transcriptional regulatory processes. ST8SIA6-AS1 has been implicated in various aspects of HCC progression, including cell apoptosis, proliferation, migration, invasion, and tumor growth and metastasis (Table 1). ST8SIA6-AS1 interacts with microRNAs in the cytoplasm, thereby modulating the stability and translatability of target mRNAs. Such interactions could affect key cellular pathways related to survival, proliferation, and metastasis. ST8SIA6-AS1 sequester miRNAs that typically suppress oncogenic mRNAs, indirectly enhancing the expression of genes that promote tumorigenicity. These findings highlight the critical role of ST8SIA6-AS1 in the molecular and functional landscape of HCC, underscoring its potential as a target for therapeutic intervention and as a biomarker for diagnosing and monitoring the progression of this malignancy.

**TABLE 1:** Functions and regulatory mechanisms of ST8SIA6-AS1 in liver cancer.

Author	Expression	Experiments	Function	Regulatory axis	Mechanism	References
Xue et al., 2023	Upregulated in HBV-associated liver cancer cell lines	*In vitro*	Cell proliferation, migration and invasion, HBV expression and replication	-	-	Xue et al. (2023)
Feng et al., 2023	Upregulated in HCC cell lines	*In vitro* *In vivo*	Cell proliferation, invasion, migration and apoptosis; Tumor growth	miR-142-3p/HMGA1	ceRNA	Feng et al. (2023)
Mou et al., 2022	Upregulated in HCC cell lines	*In vitro*	Cell proliferation, migration and apoptosis	miR-651-5p/TM4SF4	ceRNA	Mou and Ding (2022)
Zhang et al., 2021	Upregulated in HCC cell lines	*In vitro*	Cell proliferation, migration and invasion	miR-142-3p	ceRNA	Zhang et al. (2021b)
Zhang et al., 2021	Upregulated in HCC cell lines	*In vitro*	Cell migration and invasion	miR-338-3p/MEPCE	ceRNA	Zhang et al. (2021a)
Kuai et al., 2021	Upregulated in HCC cell lines	*In vitro*	Cell proliferation, migration and invasion	miR-338-3p/NONO	ceRNA	Kuai et al. (2021)
Zhang et al., 2020	Upregulated in HCC cell lines	*In vitro* *In vivo*	Cell proliferation, migration and invasion; Tumor growth	miR-129-5p/DCAF4L2	ceRNA	Zhang et al. (2020)
Qin et al., 2020	Upregulated in HCC cell lines	-	-	-	-	Qin et al. (2020)
Luo et al., 2020	Upregulated in HCC cell lines	-	-	-	-	Luo et al. (2020)
Li et al 2020	Upregulated in HCC cell lines	*In vitro* *In vivo*	Cell proliferation, migration, invasion, apoptosis; Tumor growth and metastasis	miR-5195-3p/HOXB6	ceRNA	Li and Jiang (2020)
Fei et al., 2020	Upregulated in HCC cell lines	*In vitro*	Cell proliferation, apoptosis	miR-4656/HDAC11	ceRNA	Fei et al. (2020)

HBV: hepatitis B virus, HCC: hepatocellular carcinoma, ceRNA: competing endogenous RNA.

## 3 Biological functions of ST8SIA6-AS1 in HCC

**FIGURE 1:**
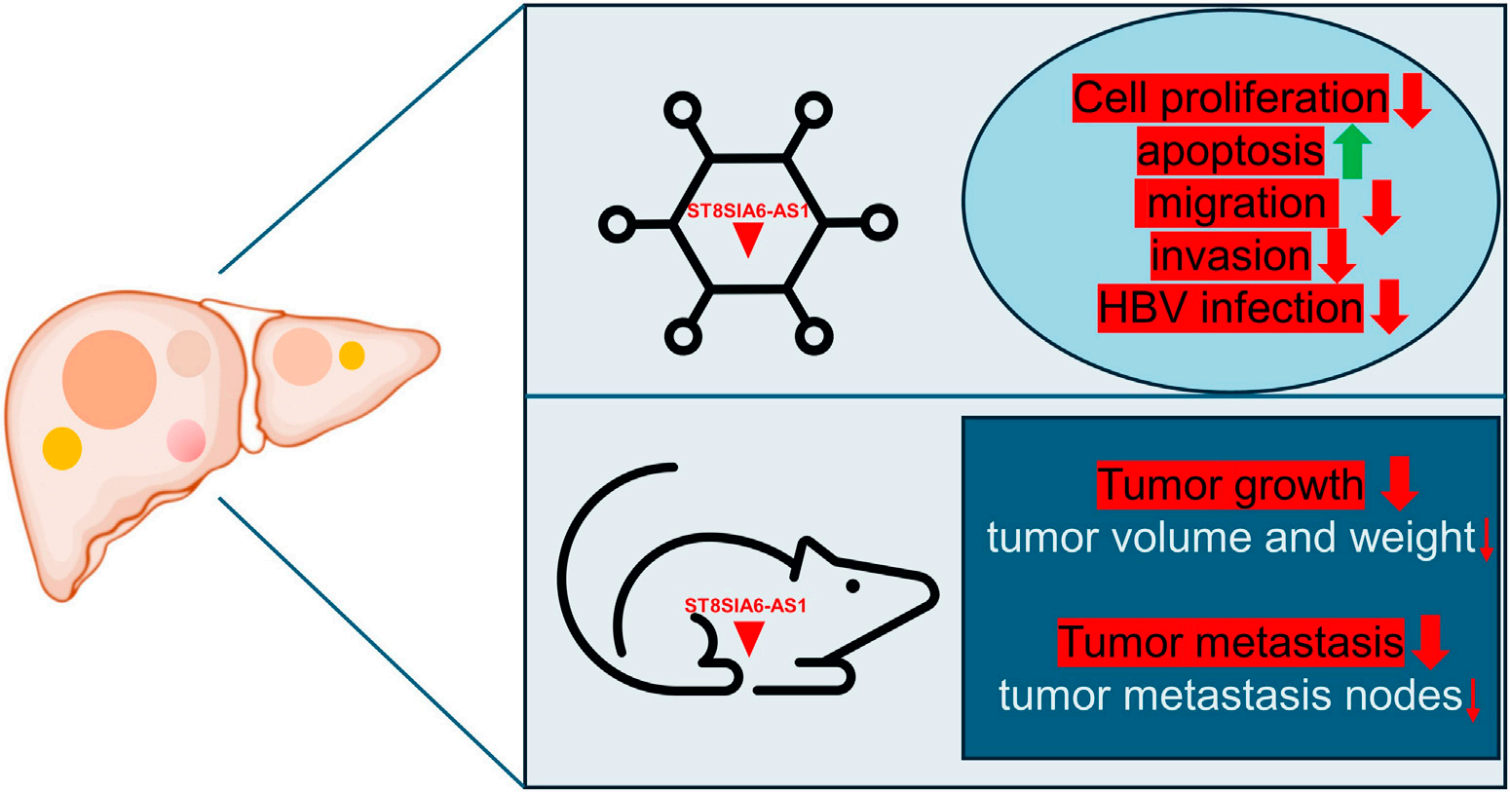
Impact of ST8SIA6-AS1 knockdown on liver cancer progression: insights from cell functional assays and xenograft tumor model. Silencing ST8SIA6-AS1 reduces liver cancer cells’ proliferative, migratory, and invasive abilities *in vitro*, and their susceptibility to HBV infection, while increasing apoptosis. *In vivo*, ST8SIA6-AS1 knockdown significantly inhibits both tumor growth and metastasis.

ST8SIA6-AS1 plays a crucial role in modulating cellular processes vital to cancer progression. Research consistently demonstrates that knocking down ST8SIA6-AS1 in liver cancer cells leads to decreased cell proliferation and increased apoptosis (Table 1). Moreover, silencing ST8SIA6-AS1 significantly diminishes the proliferative, migratory, and invasive capabilities as well as HBV infection of liver cancer cells *in vitro* and impedes tumor growth and metastasis *in vivo* (Table 1). The functions of ST8SIA6-AS1 in HCC are explored through both *in vivo* and *in vitro* experiments (Figure 1). These findings underscore the oncogenic role of ST8SIA6-AS1 in promoting the survival and expansion of liver cancer.

### 3.1 Modulation of cellular proliferation

One of the key functions of ST8SIA6-AS1 in liver cancer is the modulation of cellular proliferation. Experimental evidence indicates that ST8SIA6-AS1 substantially affects the growth dynamics of liver cancer cells (Fei et al., 2020; Li and Jiang, 2020; Zhang et al., 2020; Zhang Y. et al., 2021; Kuai et al., 2021; Mou and Ding, 2022; Feng et al., 2023; Xue et al., 2023). Using siRNA or shRNA-mediated knockdown results in a marked decrease in cell proliferation rates across various liver cancer cell lines, as demonstrated by assays such as EdU and colony formation. These results suggest that ST8SIA6-AS1 facilitates cellular growth in liver cancer, potentially by regulating gene expression involved in the cell cycle and growth signaling pathways. The capacity of ST8SIA6-AS1 to modulate proliferation highlights its potential as a target for therapeutic strategies aimed at controlling the rapid and often uncontrolled growth characteristic of malignant cells.

### 3.2 Effect on cellular apoptosis

Functional assays have revealed that the knockdown of ST8SIA6-AS1 not only impedes cellular proliferation but also alters the expression of key apoptotic regulators (Fei et al., 2020; Li and Jiang, 2020; Mou and Ding, 2022; Feng et al., 2023). Specifically, downregulation of ST8SIA6-AS1 is associated with increased expression of the pro-apoptotic protein Bax and decreased expression of the anti-apoptotic protein Bcl-2, as confirmed by Western blot analysis (Mou and Ding, 2022; Feng et al., 2023). Flow cytometry has also shown that ST8SIA6-AS1 knockdown elevates the percentage of apoptotic cells in cancer cell populations (Fei et al., 2020). These changes contribute to the heightened apoptosis observed in HCC cell lines upon ST8SIA6-AS1 silencing, indicating its role in cell cycle and apoptosis regulation.

### 3.3 Modulation of cellular migratory and invasive capabilities

The modulation of migratory and invasive capabilities by ST8SIA6-AS1 is a critical aspect of its role in liver cancer progression. Research indicates that silencing ST8SIA6-AS1 significantly curtails these capabilities in HCC cells (Li and Jiang, 2020; Zhang et al., 2020; Zhang B. et al., 2021; Zhang Y. et al., 2021; Kuai et al., 2021; Feng et al., 2023; Xue et al., 2023). This reduction is evident *in vitro*, where treated cells exhibit decreased mobility and invasiveness in standardized assays such as wound healing and transwell migration/invasion tests (Li and Jiang, 2020; Zhang B. et al., 2021; Mou and Ding, 2022; Feng et al., 2023). Notably, the knockdown of ST8SIA6-AS1 also inhibits cancer cell migration and invasion under hypoxic conditions (Zhang B. et al., 2021). These findings suggest that ST8SIA6-AS1 likely regulates a network of genes and signaling pathways that control cytoskeletal dynamics and cellular adhesion, factors essential for the metastatic spread of cancer cells. By influencing these processes, ST8SIA6-AS1 not only affects tumor growth but also plays a pivotal role in the metastatic potential of liver cancer, making it a key target for interventions aimed at limiting cancer dissemination.

### 3.4 Impact on tumor growth and metastasis

*In vivo* experiments further explore the effects on tumor growth and metastasis (Li and Jiang, 2020; Zhang et al., 2020; Feng et al., 2023). Knockdown of this lncRNA in xenograft models resulted in marked suppression of tumor growth, with observed reductions in both tumor volume and weight, demonstrating a significant tumor inhibition rate (Zhang et al., 2020; Feng et al., 2023). Additionally, studies have shown that ST8SIA6-AS1 depletion decreases metastasis nodes and downregulates proliferation markers like Ki-67 and PCNA in tissue sections from mouse models (Li and Jiang, 2020), underscoring its crucial role in tumor metastasis and growth.

### 3.5 Influence on viral oncogenesis

Additionally, ST8SIA6-AS1 also interacts with viral factors in hepatitis B virus (HBV)-infected liver cancer cells (Xue et al., 2023). Knockdown of ST8SIA6-AS1 significantly reduces HBV DNA levels, as well as the expression of HBV surface and e-antigens (Xue et al., 2023). This suggests a potential role of ST8SIA6-AS1 in modulating HBV-related oncogenic processes in liver cancer.

## 4 Molecular regulatory mechanisms of ST8SIA6-AS1 as ceRNA

ST8SIA6-AS1 influences the expression of downstream genes by participating in competitive interactions with microRNAs (miRNAs), which are integral to the competing endogenous RNA (ceRNA) network. According to the ceRNA hypothesis (Salmena et al., 2011; Ala, 2020; Xu et al., 2022), lncRNAs and mRNAs that share miRNA binding sites engage in dynamic competitive interactions for miRNA engagement, thus reciprocally affecting each other’s expression levels.

**FIGURE 2:**
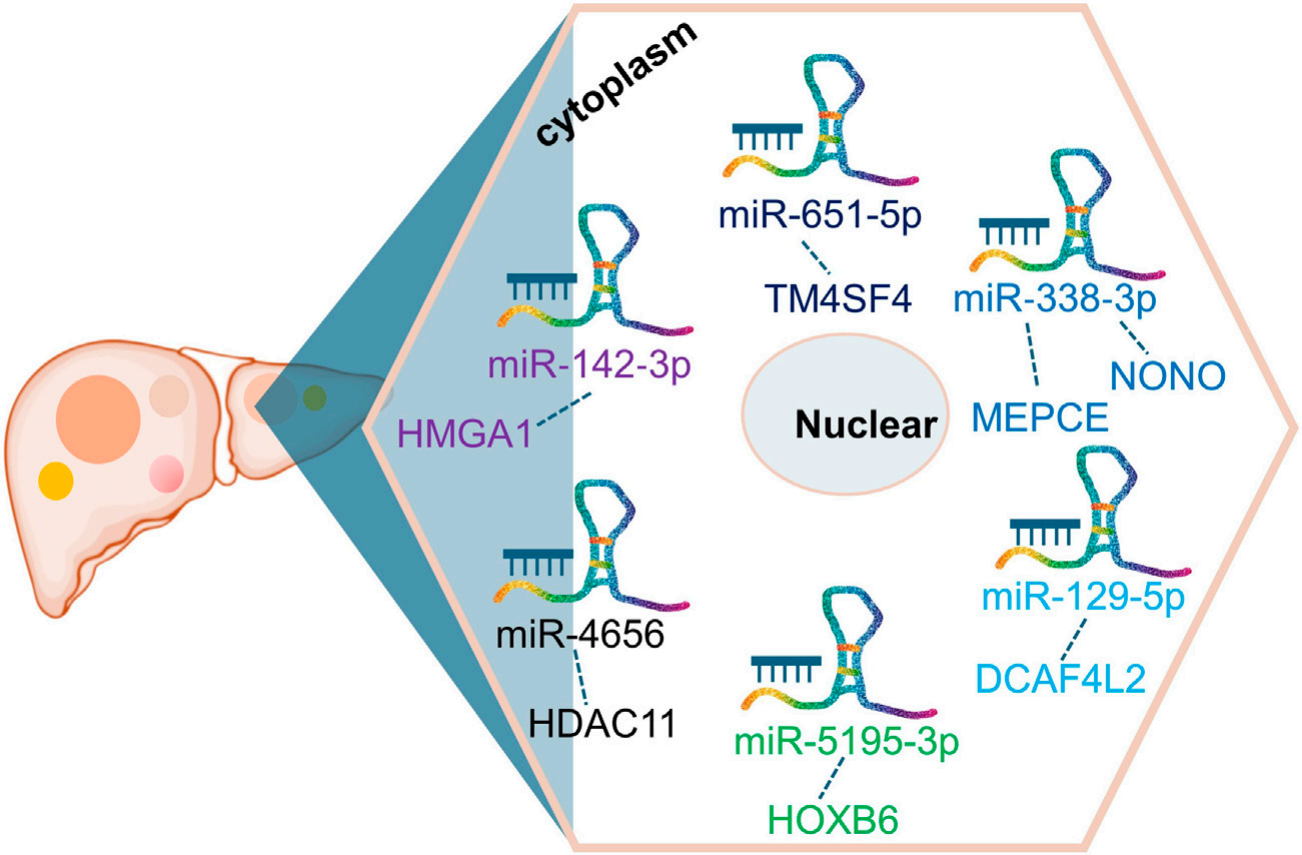
CeRNA network involving ST8SIA6-AS1 in liver cancer. ST8SIA6-AS1 competitively binds to multiple miRNAs, including miR-142-3p, miR-651-5p, miR-338-3p, miR-129-5p, miR-5195-3p, miR-4656, and miR-142-3p, leading to the upregulation of genes such as HMGA1, TM4SF4, NONO, MEPCE, DCAF4L2, HOXB6, and HDAC11 in liver cancer.

As a notable lncRNA, ST8SIA6-AS1 possesses multiple potential miRNA binding sites, enabling it to regulate various mRNAs by competing for miRNA molecules. This competitive mechanism impacts gene expression downstream, ultimately influencing liver cancer behavior and progression. In HCC (Fei et al., 2020; Li and Jiang, 2020; Zhang et al., 2020; Zhang B. et al., 2021; Zhang Y. et al., 2021; Kuai et al., 2021; Mou and Ding, 2022; Feng et al., 2023), ST8SIA6-AS1’s ability to bind competitively to several miRNAs, including miR-142-3p, miR-651-5p, miR-338-3p, miR-129-5p, miR-5195-3p, miR-4656, and miR-142-3p, facilitates the upregulation of genes such as HMGA1, TM4SF4, NONO, MEPCE, DCAF4L2, HOXB6, and HDAC11(Figure 2). For instance, Feng et al. (Feng et al., 2023) investigated the ST8SIA6-AS1/miR-142-3p/HMGA1 axis in HCC, revealing that ST8SIA6-AS1 inhibits HMGA1 expression by sponging miR-142-3p in liver cancer cells. HMGA1, known for its high expression in HCC, promotes cancer cell growth and migration (Chang et al., 2005; Andreozzi et al., 2016; Shi et al., 2022). This interaction underscores ST8SIA6-AS1’s role in enhancing HMGA1-mediated oncogenic effects in HCC, contributing to its pathogenesis. Similarly, ST8SIA6-AS1 acts as a ceRNA for miR-651-5p, suppressing HCC progression by downregulating TM4SF4 (Mou and Ding, 2022), an oncogene implicated in HCC proliferation. TM4SF4 has been reported to be highly expressed in HCC tissues, and its role in promoting cancer cell proliferation and migration has been well-documented (Li et al., 2012; Wang et al., 2013). Moreover, ST8SIA6-AS1 regulates miR-338 to upregulate MEPCE, enhancing migration and invasion in hypoxia-treated HCC cells (Zhang B. et al., 2021). The upregulation of MEPCE, a target of miR-338, is associated with increased aggressiveness in HCC under hypoxic conditions, highlighting ST8SIA6-AS1’s role in tumor progression under specific microenvironmental conditions (Zhang B. et al., 2021). Additionally, ST8SIA6-AS1 promotes HDAC11 expression by sponging miR-4656, thereby promoting HCC cell proliferation and resistance to apoptosis (Fei et al., 2020). HDAC11’s involvement in HCC progression has been noted, with its upregulation correlating with enhanced tumor cell survival and growth (Sun et al., 2018; Gong et al., 2019; Bi et al., 2021). The detailed interactions highlight ST8SIA6-AS1’s role as a ceRNA in HCC, influencing the expression of pivotal genes involved in cancer progression. Through its ceRNA activity, ST8SIA6-AS1 interacts with miRNAs to upregulate specific oncogenic factors, promoting malignant traits such as cell proliferation, invasion, and migration. This mechanism accelerates the initiation and progression of liver cancer. Further exploration of ST8SIA6-AS1’s interactions with additional miRNAs in tumor modulation holds promise for future research.

## 5 Clinical applications of ST8SIA6-AS1 in HCC

Studies have demonstrated that abnormal expression of ST8SIA6-AS1 in both HCC tissues and serum is associated with tumor progression and cancer prognosis (Table 2). The exploration of ST8SIA6-AS1 within the context of HCC has unveiled new possibilities for its clinical application, particularly as a biomarker for diagnostics and prognosis assessment. Furthermore, ST8SIA6-AS1 could serve as a therapeutic target. It may facilitate early detection of HCC, enable monitoring of disease progression, and show promise as a therapeutic intervention.

**TABLE 2:** Expression, prognostic, and diagnostic significance of ST8SIA6-AS1 in liver cancer.

Author	Expression	Significant clinical features	Survival point	Diagnosis	References
Xue et al., 2023	Upregulated in HBV-associated liver cancer tissues	-	OS	-	Xue et al. (2023)
Feng et al., 2023	Upregulated in HCC tissues	Serum AFP, lymph node metastasis, TNM stage	-	-	Feng et al. (2023)
Mou et al., 2022	Upregulated in HCC tissues	-	-	-	Mou and Ding (2022)
Zhang et al., 2021	Upregulated in HCC tissues	-	OS	-	Zhang et al. (2021b)
Zhang et al., 2021	Upregulated in HCC tissues	Histological grade, TNM stage; vein invasion	OS	-	Zhang et al. (2021a)
Kuai et al., 2021	Upregulated in HCC tissues			-	Kuai et al. (2021)
Zhang et al., 2020	Upregulated in HCC tissues	Sex, tumor number, pathologic grade	OS	-	Zhang et al. (2020)
Qin et al., 2020	Upregulated in HCC serum	AFP level, ALT level, AST level, total bilirubin level, lymph node metastasis, clinical stage	-	AUC (0.9197) Sensitivity (88.31%), specificity (74.70%)	Qin et al. (2020)
Luo et al., 2020	Upregulated in HCC tissues	-	OS	-	Luo et al. (2020)
Li et al., 2020	Upregulated in HCC tissues	-	-	-	Li and Jiang (2020)
Fei et al., 2020	Upregulated in HCC tissues	Clinical stage	-	-	Fei et al. (2020)

HBV: hepatitis B virus, AFP: α-fetoprotein, ALT: alanine aminotransferase, AST: aspartate transaminase, TNM: Tumor, node, metastasis, HCC: hepatocellular carcinoma, OS: overall survival, AUC: area under the curve.

### 5.1 ST8SIA6-AS1 is a potential diagnostic marker for HCC

The early detection of HCC remains a significant challenge (Parikh et al., 2020; Wang and Wei, 2020). Current diagnostic biomarkers for HCC, such as alpha-fetoprotein (AFP), has limited sensitivity and specificity for early detection (Pai and Parikh, 2024). This gap underscores the need for more reliable biomarkers. In recent years, multiple blood-derived lncRNAs have been identified as potential diagnostic biomarkers for several tumors (Anfossi et al., 2018; Badowski et al., 2022; Khawar et al., 2022; Toden and Goel, 2022), including liver cancer (Huang Z. et al., 2020; Shi et al., 2024; Tian et al., 2024). Among these, ST8SIA6-AS1 stands out as a promising diagnostic marker for HCC due to its distinctive expression in HCC tissues and detectability in serum (Qin et al., 2020). ST8SIA6-AS1 was significantly upregulated in serum samples from HCC patients *versus* healthy controls (Qin et al., 2020). The level of ST8SIA6-AS1 was significantly positively correlated with clinical parameters such as AFP, ALT, AST, and total bilirubin levels, which are important and common indicators of liver function (Sharma, 2022; Tamber et al., 2023). Elevated serum levels of ST8SIA6-AS1 suggest greater liver damage. Additionally, higher serum levels of ST8SIA6-AS1 indicate advanced tumor stages and increased metastatic potential (Qin et al., 2020), highlighting its role in monitoring the HCC progression. In diagnostic performance assessments, ST8SIA6-AS1 has shown high sensitivity and specificity in distinguishing HCC from healthy controls (Qin et al., 2020). ROC curve analyses have demonstrated that ST8SIA6-AS1 alone has a higher diagnostic accuracy than AFP, and when used in combination with AFP, the accuracy improves further (Qin et al., 2020). These findings suggest that ST8SIA6-AS1 could significantly enhance the diagnostic landscape of HCC, particularly in settings where AFP alone is insufficient.

### 5.2 ST8SIA6-AS1 is a promising prognostic indicator for HCC

ST8SIA6-AS1 has emerged as a biomarker for prognostic evaluation in HCC (Luo et al., 2020; Zhang et al., 2020; Zhang B. et al., 2021; Zhang Y. et al., 2021), including in cases related to hepatitis B virus (HBV) infection (Xue et al., 2023). Research has consistently shown that ST8SIA6-AS1 is upregulated in HCC tissues as compared to normal liver tissues (Fei et al., 2020; Li and Jiang, 2020; Zhang et al., 2020; Zhang B. et al., 2021; Zhang Y. et al., 2021; Kuai et al., 2021; Luo et al., 2022), and this upregulation is significantly associated with more aggressive disease characteristics (Fei et al., 2020; Zhang B. et al., 2021; Feng et al., 2023), such as higher histological grades, advanced TNM stages, and increased vein invasion (Table 2), all of which contribute to a poorer prognosis. Furthermore, studies have demonstrated a significant correlation between elevated levels of ST8SIA6-AS1 and reduced overall survival rates in patients (Luo et al., 2020; Zhang et al., 2020; Zhang B. et al., 2021; Zhang Y. et al., 2021), underscoring its utility in predicting disease progression in HCC. Elevated ST8SIA6-AS1 levels are also predictive of poor outcomes in HBV-associated liver cancer (Xue et al., 2023). The consistent overexpression of this lncRNA in HCC tissues highlights its potential as a reliable indicator of prognosis, offering a valuable tool for stratifying patients based on risk and guiding more personalized treatment approaches.

### 5.3 ST8SIA6-AS1 holds promise as a therapeutic target for HCC

ST8SIA6-AS1 has emerged as a promising therapeutic target in HCC due to its pivotal role in regulating tumor proliferation, metastasis, and survival pathways (Table 1). ST8SIA6-AS1 is significantly upregulated in HCC tissues compared to normal liver tissues (Fei et al., 2020; Li and Jiang, 2020; Zhang et al., 2020; Zhang B. et al., 2021; Zhang Y. et al., 2021; Kuai et al., 2021; Luo et al., 2022). Its overexpression is strongly associated with poor prognosis HCC patients (Table 2) (Luo et al., 2020; Zhang et al., 2020; Zhang B. et al., 2021; Zhang Y. et al., 2021; Xue et al., 2023). This underscores its potential as a key focus for innovative therapeutic interventions.

Numerous studies have highlighted the critical functions of ST8SIA6-AS1 in HCC. Knockdown of ST8SIA6-AS1 has been shown to enhance apoptosis and reduce cell migration, invasion, tumor growth, and metastasis in both *in vitro* and *in vivo* models (Table 1). For instance, Mou et al. (Mou and Ding, 2022) demonstrated that silencing ST8SIA6-AS1 weakened the proliferative and migratory capacities of HCC cells. Similarly, Li et al. (Li and Jiang, 2020) reported that ST8SIA6-AS1 knockdown significantly suppressed tumor growth and metastasis in a mouse xenograft model. These findings suggest that targeting ST8SIA6-AS1 could effectively impair cancer cell viability and halt disease progression.

ST8SIA6-AS1 functions as a ceRNA, meaning it can sponge miRNAs and modulate the expression of miRNA target genes. For example, ST8SIA6-AS1 interacts with miR-5195-3p (Li et al., 2022), thereby regulating the expression of oncogenic genes such as N-cadherin, SNAIL1, β-catenin, and VE-cadherin, which are crucial for epithelial-mesenchymal transition (EMT) (Kaufhold and Bonavida, 2014; Loh et al., 2019) and tumor angiogenesis (Vestweber, 2008; Wang et al., 2022). By acting as a ceRNA, ST8SIA6-AS1 integrates into complex molecular networks that drive malignant behaviors in tumor cells, offering multiple angles for therapeutic targeting.

Emerging therapeutic strategies targeting ST8SIA6-AS1 include the development of small interfering RNAs (siRNAs) or antisense oligonucleotides (ASOs) designed to inhibit its expression. Such approaches aim to disrupt the malignant phenotype by modulating the lncRNA’s interactions with miRNAs and altering the expression of oncogenic genes. For instance, Fei et al. (Fei et al., 2020) demonstrated that siRNA-mediated knockdown of ST8SIA6-AS1 repressed cell proliferation and induced cell apoptosis in HCC cells by targeting the miR-4656/HDAC11 axis. Additionally, ASO-based therapies have shown promise in preclinical studies for silencing specific lncRNAs in cancer, offering a potential avenue for ST8SIA6-AS1-targeted treatment.

## 6 Perspectives

In our review, we have detailed the biological functions and regulatory mechanisms of ST8SIA6-AS1 within liver cancer, emphasizing its promise as both a diagnostic and prognostic marker, and as a potential therapeutic target in HCC. Current research into the role of ST8SIA6-AS1 in HCC, however, remains in its early stages. Further *in vitro* and *in vivo* studies are needed to determine how this lncRNA influences the initiation and progression of liver cancer. The exact pathways through which ST8SIA6-AS1 operates in HCC are still largely undefined, and its potential involvement in liver cancer drug resistance remains an interesting question. While known primarily for its function as a ceRNA, ST8SIA6-AS1 may also engage in other regulatory mechanisms that are critical to its role in HCC. Further investigation is necessary to explore these additional functions. Like other lncRNAs such as HOTAIR in breast cancer (Bhan et al., 2013; Portoso et al., 2017; Ma et al., 2022) and MALAT1 in lung cancer (Amodio et al., 2018; Liu J. et al., 2023; Bhat et al., 2024), ST8SIA6-AS1 could interact with chromatin-modifying proteins to influence gene expression epigenetically. Additionally, there is potential for ST8SIA6-AS1 to impact mRNA stability or translation, similar to the mechanisms observed with lncRNA PCAT6 in prostate cancer (Liu et al., 2020; Lang et al., 2021). These interactions are still unknow and need to be explored. It is conceivable that ST8SIA6-AS1 interacts with other RNAs and proteins, thus driving HCC progression and metastasis. Investigating these interactions could uncover new facets of ST8SIA6-AS1’s role in liver cancer, offering insights that could lead to novel therapeutic targets. By understanding the multifaceted roles of ST8SIA6-AS1 in comparison to other well-studied lncRNAs, researchers can better delineate its unique contributions to HCC pathology. This deeper understanding will facilitate the development of targeted therapies that could intercept these lncRNA-mediated pathways, potentially transforming HCC management and improving patient outcomes.

Serum ST8SIA6-AS1 also has emerged as a promising new biomarker for diagnosing HCC (Qin et al., 2020). This lncRNA exhibits relatively high sensitivity and specificity, reflects tumor dynamics, and correlates with disease severity (Qin et al., 2020). Integrating ST8SIA6-AS1 into routine diagnostic protocols, alongside markers such as AFP, could enhance early detection rates (Qin et al., 2020). However, research on ST8SIA6-AS1 for diagnostic purposes is currently limited to a single study, and its application in tumor diagnosis is still in its infancy. Therefore, its efficacy in diagnosing liver cancer requires large-scale, multi-center validation. Furthermore, other emerging lncRNAs, such as SNHG16 (Liu C. et al., 2023), TERC (Chen et al., 2022), and LINC00657 (Tarrad et al., 2023), have shown diagnostic potential in various body fluids, including urine and saliva. The expression of ST8SIA6-AS1 in these fluids and its diagnostic value in these matrices remain to be investigated.

In terms of prognosis, patients with high expression of ST8SIA6-AS1 have shown shorter OS (Luo et al., 2020; Zhang et al., 2020; Zhang B. et al., 2021; Zhang Y. et al., 2021). However, the relationship between ST8SIA6-AS1 and other survival indicators like progression-free survival remains unestablished. It is unclear whether ST8SIA6-AS1 can reliably predict tumor progression and recurrence. Additionally, delving into the prognostic significance of ST8SIA6-AS1 across various stages of HCC could yield valuable insights. More studies are needed to validate the prognostic value of ST8SIA6-AS1 in larger, more diverse cohorts. Future research should focus on validating these findings through larger, multi-center studies and developing standardized, cost-effective assays for ST8SIA6-AS1 to facilitate its adoption in clinical practice. This will ensure that the full potential of ST8SIA6-AS1 as both a diagnostic and prognostic biomarker is realized, offering significant improvements in the personalized treatment of liver cancer.

For the therapeutic target of ST8SIA6-AS1, the clinical application of targeting ST8SIA6-AS1 requires comprehensive studies to validate its efficacy and safety. Early-phase clinical trials are essential to assess the therapeutic potential of siRNAs or ASOs against ST8SIA6-AS1 in HCC patients. Moreover, identifying biomarkers for patient stratification could enhance the success of ST8SIA6-AS1-targeted therapies by selecting patients most likely to benefit from such treatments. Future research should focus on elucidating the precise molecular mechanisms of ST8SIA6-AS1 in HCC, optimizing delivery methods for siRNAs/ASOs, and conducting clinical trials to evaluate their therapeutic potential comprehensively. ST8SIA6-AS1 represents a compelling target for therapeutic intervention in HCC due to its significant role in promoting tumor growth and metastasis. The development of targeted therapies against ST8SIA6-AS1, such as siRNAs or ASOs, holds promise for improving patient outcomes.

## 7 Conclusion

In summary, ST8SIA6-AS1 plays a pro-tumorigenic role in the development of liver cancer and regulates malignant processes through ceRNA networks. This lncRNA may serve as both a prognostic and diagnostic marker and holds significant promise as a target for the development of targeted therapies in HCC. By modulating this lncRNA, future therapeutic strategies may effectively disrupt the complex molecular interactions associated with tumor progression, offering a novel approach for improving HCC treatment outcomes.
